# Aflatoxin-Contaminated Nut Separation by Applied Machinery and Processing Stages in Fresh Pistachio Processing Plant

**DOI:** 10.3389/fmicb.2017.02404

**Published:** 2017-12-13

**Authors:** Ebrahim Hadavi, Hamid Feizi, Behrouz Gheibi

**Affiliations:** ^1^Department of Horticulture, Karaj Branch, Islamic Azad University, Karaj, Iran; ^2^Horticulture Department, Ministry of Agriculture, Tehran, Iran

**Keywords:** aflatoxin B1, adhering hull, HACCP, early split, post-harvest

## Abstract

In pistachio nuts, aflatoxin is mainly concentrated in the green hull-cracked nuts in the orchard even prior to harvest. However, during the hull-removal process, the hulls of all nuts including the cracked, identifiable nuts are removed causing an important characterization criterion of the contaminated nuts, to be lost. This, in turn, makes it harder to detect the contaminated nuts. However, during the processing stage, there is a good possibility of sorting the contaminated nuts based on their other inherent features, such as specific gravity and stain on the hard shell. In this study, we aimed to explore the effect of applied processing machinery and related sequences in the aflatoxin flow during post-harvest processing and the possible ways of reducing aflatoxin contamination in the Iranian pistachio industry. We planned a systematic sampling from the main and reject streams in two major prevailing processing methods, namely, wet and dry. In the dry processing method, the reject streams such as the adhering-hull rejects, air floaters, and manual hand pick-outs from the final inspection were found to be significantly more contaminated than the main stream. However, in the wet processing method, the input stream was considered highly contaminated. Among the sorting stages, only the adhering-hull rejects of the sinkers were considered more contaminated than the main stream. A contamination removal index was developed and applied to compare the effectiveness of the processing procedures. The water flotation tank was deemed responsible for 52.5% of aflatoxin contamination removal compared to 2.1% in the air flotation system. More effective sorting was achieved by the adhering-hull remover, which was preceded by a rubber-drum huller instead of the metal-drum type. Thus, by combining the most effective techniques of aflatoxin removal, an improved and more efficient method may be designed for pistachio processing plants.

## Introduction

In pistachio nuts, aflatoxin contamination is found to be heterogeneous and concentrated in a few nuts. For example, Schatzki and Pan (1996) estimated that there is 1 highly contaminated nut per 10,000 to 1,000,000 nuts, and Sommer et al. (1986) estimated that there is 1 contaminated nut per 25,000 nuts. This distribution pattern may be considered as an advantage if we develop screening methods that can effectively identify aflatoxin-contaminated nuts (Hadavi, 2005). Many studies have been conducted to understand the distribution pattern of aflatoxin-contaminated nuts in the orchard (Doster and Michailides, 1994a,b,c,d, 1995; Sommer et al., 1976; Thomson and Mehdy, 1978), after harvest (Schatzki, 1995b), in the processing streams (Schatzki and Pan, 1996), and in the pistachio lots (Schatzki, 1995a). An improved understanding of the distribution of aflatoxin-contaminated nuts is necessary both to develop proper screening methods for decreasing the contamination level and also to improve the applied sampling methods for detecting the contamination level of aflatoxin.

After processing the fresh pistachios by the processors, the resulting dried pistachios are subjected to extensive sorting that is usually performed by hand or by employing visual or light reflectance electronic sorting techniques to remove defective nuts or aflatoxin-contaminated nuts (Boutrif, 1998). Campbell et al. (2003) stated that intensive sorting is already being performed for quality, resulting in some process streams among which aflatoxin is associated with only a few of low-volume ones. The removal of contaminated nuts in the relevant streams plus hand sorting, may result in reduced aflatoxin levels.

However, the optimization of fresh pistachio processing has not been studied enough. This is illustrated by the vast diversity that exists among the pistachio processors and applied machinery as well as the differences in process flow design. Indeed, parallel methods and machinery with different types of logic behind their design are used for the same sorting objective. Clearly, if we understood how aflatoxin flows through the different fresh pistachio-processing machinery, we may have an idea of how to improve the design in terms of maximum aflatoxin removal. As a result, this could decrease or even eliminate the need for the most expensive processing steps by automated sorters.

Worldwide, the “wet processing” method was more prevalent before public awareness of the aflatoxins risk in pistachio nuts. The incoming fresh pistachios (input stream), are hulled and then enter a water tank. The floating nuts are later expelled from the processing stream (main stream). As the water in these floating tanks was often recycled, the European Union (EU) experts suggested the practice to be discontinued on the basis that it was unsanitary. Consequently, the local machine manufacturers developed an air flotation table, which was substituted for the water flotation tank and the new setup was named “dry processing.” The subsequent processes of sorting like removal of adhering-hull pistachios (the nuts which still hold the hull after the hulling process) remain more or less similar.

In this study, we aimed to evaluate the existing processing machinery and the main process flow design for their effectiveness in reducing aflatoxin-contaminated nuts.

## Materials and Methods

### The Research Context

This study was conducted in two selected fresh pistachio processing plants which were managed under the supervision of a hazard analysis and critical control point (HACCP) pilot plan. As a result, the transport from the orchard was conducted to ensure timely harvest and uninterrupted transfer to the processor. The processors were located in Kerman province, approximately 100 km apart, therefore, the processed pistachio lots were different. Sampling points were selected to reflect the processing stages thought to be most relevant to the aflatoxin flow. The experiment was conducted during the 2004 harvest season.

### Processing Methods

**FIGURE 1:**
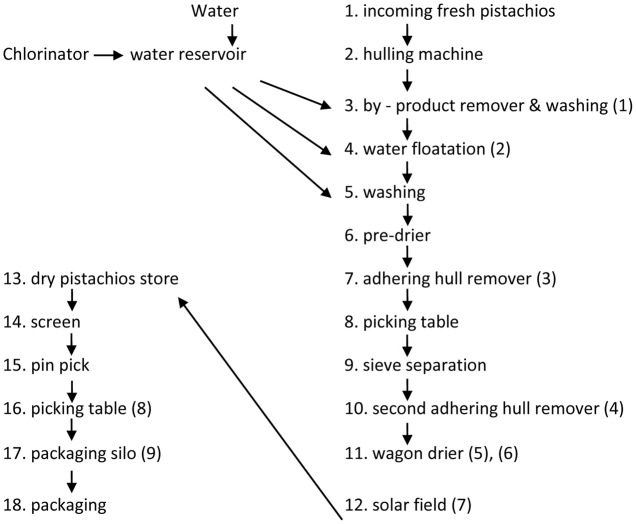
Flow diagram of wet Processor. The numbers in brackets correspond to the sampling points. (1) input stream; (2) water floaters; (3) first rejects of sinker’s adhering-hull; (4) second adhering-hull rejects; (5) sinkers before the dryer; (6) partially dried sinkers; (7) sinkers after sun drying; (8) manual rejects from the final inspection; (9) the final lot.

**FIGURE 2:**
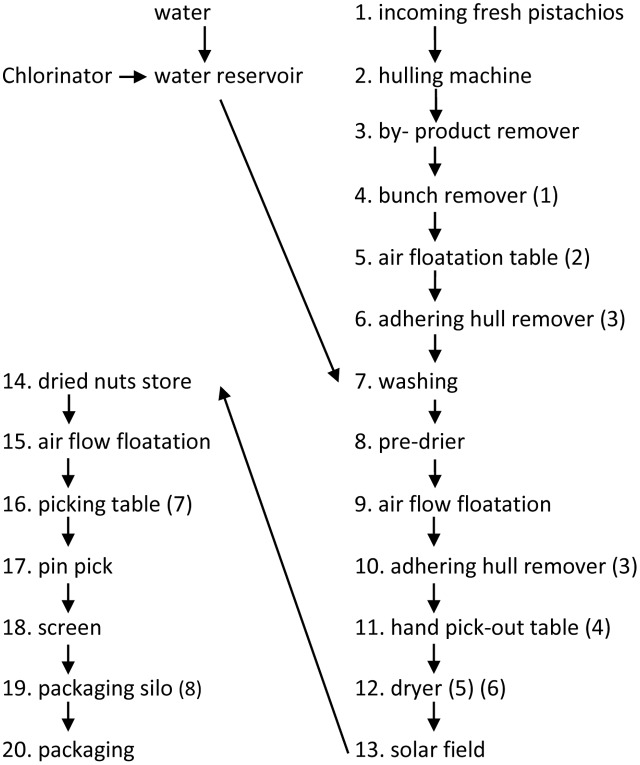
Flow diagram of dry Processor. The numbers in brackets correspond to the sampling points. (1) input stream; (2) air floaters; (3) adhering-hull rejects; (4) hand rejects; (5) before drying; (6) after drying; (7) manual rejects from the final inspection; (8) the final lot.

In this study, we compared the two major prevailing processing methods: “wet processing” that is characterized by the use of a water floatation tank after hulling the nuts (**Figure 1**), and “dry processing” that is characterized by substitution of an air flotation table instead of the water floatation tank (**Figure 2**).

### Sampling Procedure for Aflatoxin Measurement

To ensure similar sampling procedure among the processors, a guideline was established, and personnel were trained accordingly. Sampling was planned in such a way, as to avoid any disruption of the usual processing practices. Nine wet processing and eight dry processing points were selected for sampling. Imported lots were owned by the traditional clients of each processor and each originated from a known parcel of the orchard as the production unit. A uniform and easy to conduct sampling was developed to lessen the related error. Sampling was conducted continuously during the processing of each pistachio lot. At each sampling point, an incremental sample was collected every 15 min using a 500-mL container (containing 170–250 g pistachios depending on the moisture content) and finally weighed to constitute an aggregate sample of ≈ 2 to 3 kg for a typical 1,500 kg lot. As a consequence, the final sample weight depended on the lot size. A total of 20 lots, with a total dry weight of 27,070 kg (average 1,353 kg) were processed by the dry method, and 31 lots with a total dry weight of 46,858 kg (average 1,511 kg) were processed by the wet method. For an average lot, it took at least 3 h to pass through each sampling point, which meant that over a 15 min sampling interval, roughly a total of 12 samples were pooled at each monitored processing stage. Considering eight sampling points in the dry processor and nine in the wet processor, a total of 426 aggregate samples were collected and analyzed for aflatoxin content. The final sample was collected from the final processed product of each lot based on the (European Commission [EC], 1998) Directive 98/53/EC as it was achieved in 2004.

The sampling points in the wet and dry processor are described in **Figures 1**, **2**, respectively.

### Aflatoxin Measurement

Homogenized water slurries of pistachio nut samples were prepared by adding 1.5 L water to every kilogram of ground pistachio nut using ultra-turrax. Based on the moisture content reported by the processor, the internal water content of the samples was deducted from added water for the nuts collected before drying. All the samples were analyzed for aflatoxin content by an independent accredited laboratory based on the AOAC Official Method 999.07 “Immunoaffinity Column Cleanup with Liquid Chromatography Using Post-Column Bromination for Determination of Aflatoxins in Peanut Butter, Pistachio Paste, Fig Paste, and Paprika Powder” as reported by Stroka et al. (2000). For quantification, a reverse-phase high-performance liquid Chromatography (Waters, Milford, MA, United States) with post-column derivatization using kobra cell system and 2475 fluorescence detector was used. Limit of detection (LOD) and limit of quantification (LOQ) for aflatoxin B1 (AFB1) were 0.03 and 1 μg/kg pistachio nuts. Extraction recovery for AFB1 was between 83 and 105%. As AFB1 was the key detected aflatoxin in pistachio lots, we excluded the data of other aflatoxins for simplicity (Supplementary Tables S1, S2).

### Aflatoxin Removal Assessment

An aflatoxin removal effectiveness index at each processing stage was developed. The relative mean share of the reject streams from the total stream of the sampling point (per 1000) was obtained from the processor. This share was multiplied by the mean contamination of the rejection stream in the same observation point to give the contamination removal index (CRI). The index was used to compare the effectiveness of the machinery as well as the processing stage within and between the processors.

$$\frac{\text{reject weight }\left(\text{g}\right)}{\text{input weight }\left(\text{kg}\right)}\times \frac{\text{aflatoxin B1 in reject }\left(\text{ng}\right)}{\text{reject weight }\left(\text{g}\right)}\equiv \frac{\text{aflatoxin B1 in reject }\left(\text{ng}\right)}{\text{input weight }\left(\text{kg}\right)}=CRI$$

### Data Preparation and Analysis

Because of the inherent variation in the aflatoxin content of the samples, the aflatoxin data transformation was tested and the natural logarithm (Ln) deemed suitable. Therefore, to make the logarithmic data transformation possible, we substituted 0.03 (LOD) instead of zero for the none detected (nd) results. The transformed data were analyzed using the SPSS software, and the means were compared by Duncan’s test at 0.05 level of alpha (α) (Version 16.00, SPSS, Inc.).

## Results

### Aflatoxin Contamination in Reject Streams

**FIGURE 3:**
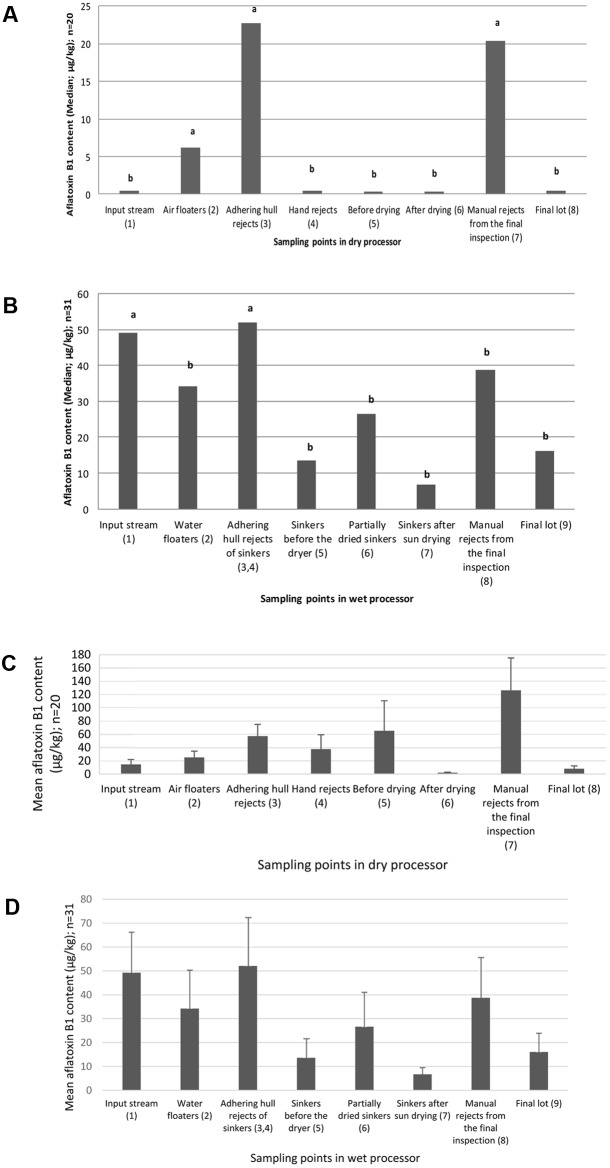
**(A)** Median of the aflatoxin content in aggregate samples collected from designated sampling point in the dry processed pistachio stream. **(B)** Median of the aflatoxin content in aggregate samples collected from designated sampling points in the wet processed pistachio stream. The mean comparison has been carried out on natural logarithm (Ln) transformed data of aflatoxin content (μg/kg). Values with similar letters are not significantly different according to statistical analysis (*p* < 0.05; see section “Material and Methods”); and the adjacent numbers are sample count (n). **(C)** Mean and standard error of the aflatoxin content in aggregate samples collected from designated sampling point in the dry processed pistachio stream. **(D)** Mean and standard error of the aflatoxin content in aggregate samples collected from designated sampling points in the wet processed pistachio stream. Error bars represent standard error. The numbers in brackets correspond to the sampling points listed in caption of **Figures 1** and **2**. In the wet processor, the first and second rejects of adhering hull nuts data are pooled.

As seen in **Figure 3A**, the adhering-hull rejects, air floaters, and the manual hand pick-outs from the final inspection contained significantly more aflatoxin than did the rest of the sampling points in dry processing (*p* < 0.05). However, only the adhering-hull rejects and the input stream nuts were significantly more contaminated than the rest of the sampling points of the wet processor (*p* < 0.05; **Figure 3B**).

### Assessment of Effectiveness of Rejection Streams by the Use of the CRI Index

A comparison was made among the different stages in the two processors by using the CRI index instead of the contamination level. Based on a substantially lower CRI index value, achieved by the (air) gravity table (**Table 1**), it is suggested that the air gravity table was less efficient in removing contamination when compared to the water flotation tank. The water flotation tank removed 46.3% of the total contamination while the air flotation method removed only 2.1%.

**Table 1:** Comparing the effect of selected reject streams on reduction of aflatoxin contamination between wet and dry pistachio processing lines.

	Processing stage			
		
Processing type	Water flotation tank	Gravity table(air flotation)	Adhering-hull remover	Manual handpick-outs from final inspection	Total
Wet processing	68.5 ± 32.3	-	31.2 ± 12.2	48.4 ± 21.1	148.1
	20%^‡^		6%	8%	
	(46.3%)^∗^		(21%)	(32.6%)	(100%)
Dry processing	-	2.5 ± 1	34.3 ± 10.7	82.0 ± 31.7	118.8
		1%	6%	6.5%	
		(2.1%)	(28.9%)	(69%)	(100%)


*^†^CRI = Mean Contamination Removal Index ± Standard Error of Mean; defined as the estimated removal of aflatoxin contamination in ng, per kg of input stream by the processing stage (ng/kg). It is calculated by multiplying weight of each reject stream in its contamination level as described in M&M. ^‡^Relative mean share of the reject stream from main processing stream as reported by processors. ^∗^Relative share from the total CRI.*

As could be seen in **Figure 1**, where the wet processor used two sets of adhering-hull removers, only a 21% reduction in the total CRI is evident. On the other hand, a single adhering-hull remover reduced the contamination by 28.9% of the total CRI in the dry processing line (**Table 1**).

As denoted by the CRI value, the efficiency of the dry pistachio inspection belt in the dry processor was near 1.7-fold higher than in the wet processor.

## Discussion

### The Sampling Plan Efficiency

We noted a considerable variation in aflatoxin levels in the samples drawn from all streams (**Figure 3**). This could be due to the inherent difference between the origin of the nuts, the cultural practices, as well as the cultivar itself. Therefore, to achieve a more dependable estimation of aflatoxin content in the main streams of processing pistachio nuts, a higher frequency of sampling or a larger subsample size seems necessary.

### The Difference in Contamination Level in the Input Streams

The results suggested more aflatoxin content in the input stream of the wet processor. However, in the dry processor, any significant difference between contamination level of the input stream and the final export lot was lacking (*p* > 0.05). Given the long distance between the processors, we can expect input from different orchards therefore, the observed difference between the contamination levels of processors may be considered natural. However, for an input stream, given that for a typical lot of 1,500 kg [fresh weight (FW)], only 12 × 0.5 = 6 L by estimated weight of 1.5 kg were collected and analyzed to assess its contamination, we might realize that it may not have been a valid estimate of the contamination level. In order to yield a more dependable assessment of the contamination input from orchard to the processing unit, elaboration of a more sophisticated sampling method may need to be developed. It seems reasonable to use a more frequent and/or larger sampling size for a representative contamination estimation for the input stream, as the largest stream by throughput. However, the final estimated lot was sampled based on EU regulation, which should make it more dependable than that from the input stream. Yet, for the reject streams, the situation is different; these samples often included a notable part of the reject stream, which makes the estimated aflatoxin level more representative. In fact, in some sampling points, for example “air floaters,” the reject sample included a large share of the reject stream.

### The Effect of Processing Layout and Machinery on the Contamination Removal

The CRI index is a more reliable tool for estimating the actual contamination outflow because it takes the weight of the reject stream into account. For instance, considering there are more contaminated nuts among the air floaters compared to those at some other sampling points, air floaters would seem as an important reject stream (**Figure 3A**). However, by looking at **Table 1**, we found that air floaters constituted of only a 1% share of the main processing stream which yields a much lower CRI than do the other reject streams. This is because the CRI is reflecting the amount of AFB1 weight that is removed by each reject stream per unit of processed pistachio, which is much more informative than the AFB1 concentration in the reject stream. Therefore, CRI would be a more reliable tool in assessment of the sorting steps in term of AFB1 control, rather than of the aflatoxin contamination level. This would enable us to keep an eye on the contamination flow in the sorting process more realistically. As shown in **Table 1**, the total CRI value in wet processing (148.1 ng/kg) was on par with the dry processor (118.8 ng/kg). This suggests that, despite the difference between some sampling points, the overall effectiveness in terms of AFB1 removal was more or less similar by both processing methods. The share of contaminated lots (>8 μg/kg of AFB1) from total final export lots was 3 out of 31 lots in dry processing (10%) and 5 out of 20 lots in wet processing (25%), which were considered as “reject” based on the EC regulation 165/2010 (European Commission [EC], 2010). However, when considering the close CRI values, it seems improper to justify this difference by applied processing method. As a result, there remains a possibility for the presence of an intrinsic difference in the contamination levels between the harvested orchards. This makes sense when taking the distance between the processors into account, which were more than a 100 km apart, although, still in the same production area of Kerman province.

The CRI comparison suggested the water flotation tank to be more effective than the air flotation method in removing aflatoxin-contaminated nuts. Nevertheless, in the water flotation method, a standard separation was obtained based on the difference between densities of water and pistachio. In comparison, the air flotation method separates erratically due to the momentary changes in air flow influenced by the arbitrary movement of the nuts. In addition, the operators could adjust the machine to reduce the reject rate, which could make it more attractive to the lot owner. In conclusion, it is recommended to consider using the water flotation tank as the standard method for processing pistachio nuts.

### The Possible Role of Hulling Technique in Aflatoxin Contamination Removal

Earlier investigations have shown that the early split nuts have a tendency to hold their hulls during the hulling process (Pearson et al., 1996) and are also the main carriers of the aflatoxin in pistachio nuts. Therefore, these hull-adhering early split pistachios are the important carriers of aflatoxin in the pistachio processing streams. In our experiment, we observed a CRI value of 34.3 ng/kg by a single adhering-hull remover in the dry processor. However, two sets of the same machine were in use in the wet processor, which together yielded a CRI value of 31.2 ng/kg. This implies that the machine did better in the dry processor. Further investigations revealed that the wet processor used a metal-drum huller, while the dry processor employed a rubber-drum huller. Furthermore, the steel drums perform hulling by a plurality of projections on the drum surface (Volk, 1984), which dehull the nut mainly by shear stress (Shamsi et al., 2011). Whereas, in the rubber-type huller, the friction strips made from a resilient material-like rubber do the hulling by sheer friction (Nakhei-Nejad, 2002). As the rubber drums perform the hulling by surface friction, we could expect a higher possibility for the adhered hulls to remain adhered on the nuts thereby creating a high possibility for such nuts to be identified and rejected by subsequent adhering-hull remover. This could have been the case in the dry processor, which used a rubber-drum huller. As a result, the higher amount of contamination in the rejects from the subsequent adhering-hull remover seems natural. In fact, this combination could drive a key group of highly contaminated pistachios to find their way out of the main pistachio stream. Conversely, in a metal-drum huller, we could expect a weaker discrimination between this group and the rest of the nuts. This is attributed to the more aggressive hulling mechanism in the metal drum huller which could forcibly remove the hull of some adhering hulls that would otherwise retain their hulls by use of a friction-based rubber drum huller. Therefore, these originally adhering hull pistachios, that are now hulled by the metal drum huller could pass the adhering hull removal stage(s) and remain in the main stream causing its contamination level to elevate. Hence, based on much higher CRI attained by the “rubber drum huller/adhering hull remover” combination, we deem it as more effective compared to the “metal drum huller/adhering hull remover” combination.

### The Importance of Human Resource Management

The observed higher efficiency of aflatoxin removal by hand sorting in the dry processor as marked by a higher CRI in the dry pistachio inspection belt (**Table 1**), could be attributed to the higher number of workers combined with the lengthier inspection belt and slower produce flow rate. However, this remains an educated guessing and more data is needed to make a conclusion.

## Conclusion

The results reveal the need for process flow evaluation and its role in the design of processing flowcharts that could remove more aflatoxin from the pistachio processing streams. Based on this study, to improve the efficiency of removing aflatoxin- contaminated nuts from the pistachio processing streams, we recommend including the rubber drum huller, water flotation tank, and an adhering-hull remover in the processing design. However, further study with this approach is needed to fine-tune the processing layout and machinery in terms of aflatoxin reduction.

## Author Contributions

EH designed the experiment and wrote the manuscript. HF helped during conduction of the experiment and collecting the data. BG helped in conducting the experiment.

## Conflict of Interest Statement

The authors declare that the research was conducted in the absence of any commercial or financial relationships that could be construed as a potential conflict of interest.
